# Supplementary material for: Angelica sinensis polysaccharide as potential protectants against recurrent spontaneous abortion: focus on autophagy regulation
Source: Front Med (Lausanne). 2025 Jan 15;12:1522503. doi: 10.3389/fmed.2025.1522503 (PMC11774876; doi:10.3389/fmed.2025.1522503)
Supplement: Supplementary material S1 — The main instruments used during the LC-MS process, along with their models/specifications and manufacturers. [file Supplementary_file_1.zip › Metabolomics sequencing data FC1.2/8.代谢通路分析/Exposure_Ctrl/all/代谢通路富集图-p小于0.05.pdf]

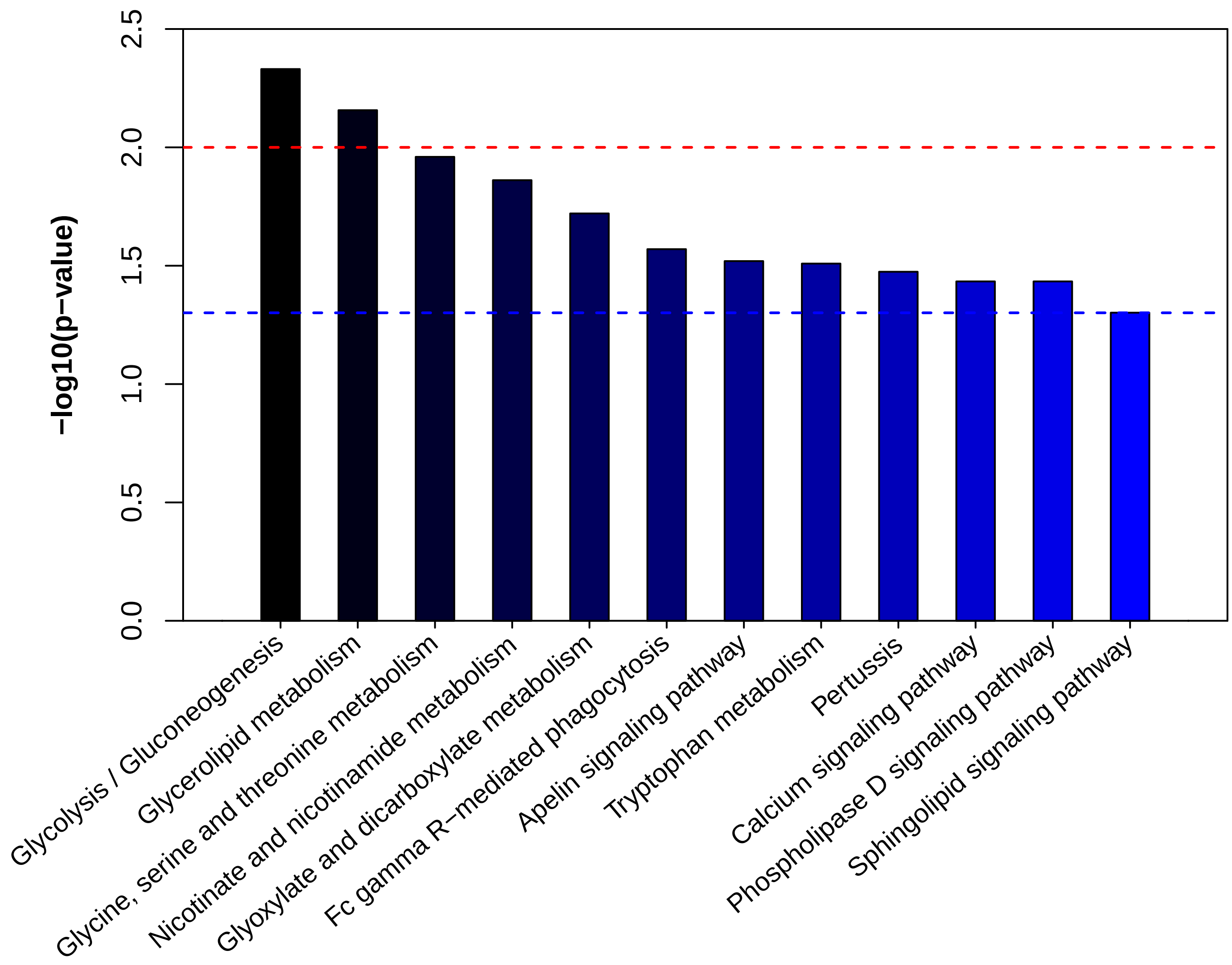

−log10(p−value)
0.0
0.5
1.0
1.5
2.0
2.5
Glycolysis / Gluconeogenesis
Glycerolipid metabolism
Glycine, serine and threonine metabolism
Nicotinate and nicotinamide metabolism
Glyoxylate and dicarboxylate metabolism
Fc gamma R−mediated phagocytosis
Apelin signaling pathway
Tryptophan metabolism
Pertussis
Calcium signaling pathway
Phospholipase D signaling pathway
Sphingolipid signaling pathway
